# P-2347. Prolonged Mpox Infections in California, May 2022 – March 2023

**DOI:** 10.1093/ofid/ofae631.2499

**Published:** 2025-01-29

**Authors:** Samuel H Schildhauer, Kayla Saadeh, Robert Snyder, Eric C Tang, Alyssa Newman, Jessica Watson, Deanna A Sykes, Philip Peters, Kathleen Jacobson, Kelly A Johnson

**Affiliations:** California Department of Public Health, San Francisco, CA; California Department of Public Health, San Francisco, CA; California Department of Public Health, San Francisco, CA; Sexually Transmitted Diseases Control Branch, Richmond, California; California Department of Public Health, San Francisco, CA; California Department of Public Health, San Francisco, CA; California Department of Public Health, Office of AIDS, Sacramento, California; California Department of Public Health, San Francisco, CA; CDPH, Richmond, California; California Department of Public Health & University of California San Francisco, San Francisco, California

## Abstract

**Background:**

Clinical mpox infections typically last from 14-28 days, with viral DNA in lesion samples detectable for up to 21 days. However, prolonged infections, with symptoms or repeat positive tests lasting > 21 days, have been occasionally documented, but not fully characterized.

**Table 1:**
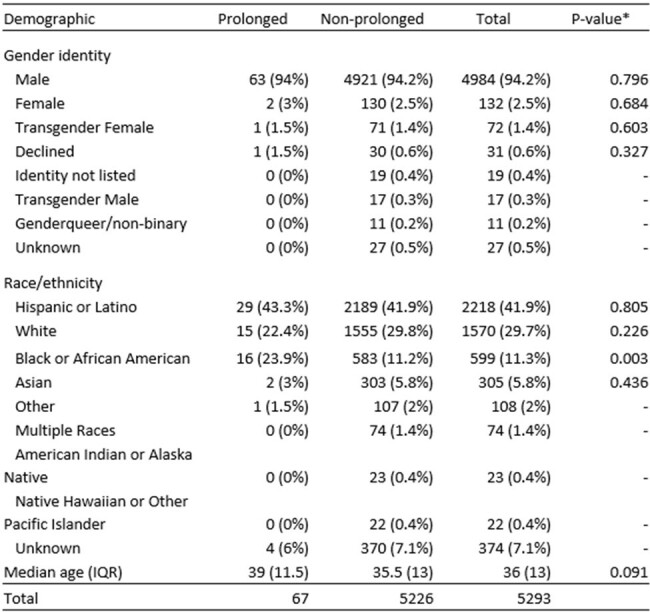
Demographic characteristics of prolonged mpox infections in California, May 12, 2022 – March 7, 2024, n = 5293

* Pair-wise comparisons were conducted between prolonged vs non-prolonged for each demographic factor using Fisher’s Exact Test. A Wilcox Test was used to compare median age between prolonged vs non-prolonged.

**Methods:**

We defined prolonged mpox infections as those with > 28 days between either two mpox virus (MPXV) polymerase chain reaction (PCR) positive tests, or > 28 days between self-reported symptom onset and a subsequent positive MPXV PCR. We then used data from California’s reportable disease system to compare prolonged and non-prolonged mpox infections from 05/12/22-03/7/23 by demographics, HIV/immunocompromised status including CD4 count among people with HIV (PWH), and mpox vaccination status. Fisher’s exact tests for pairwise comparisons were performed in R 4.3.2.

**Table 2:**
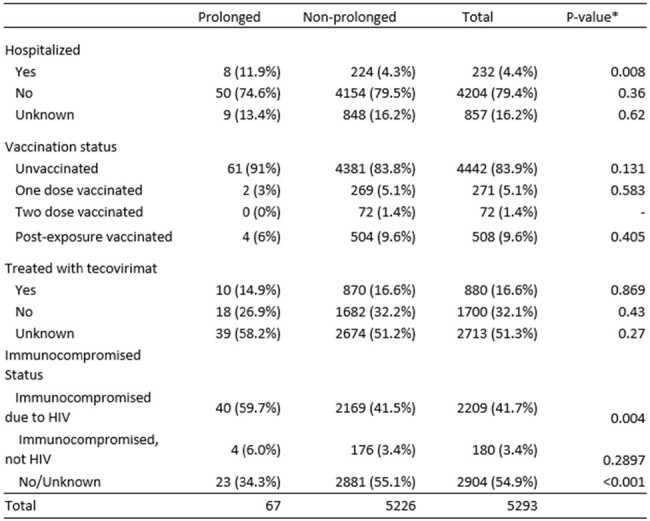
Clinical characteristics of prolonged mpox infections in California, May 12, 2022 – March 7, 2024, n=5,293

* Pair-wise comparisons were conducted comparing prolonged to non-prolonged mpox infections for each clinical factor using Fisher’s Exact Test.

**Results:**

Our analysis included 5,293 mpox infections, of which 67 (1.3%) were prolonged. Compared with non-prolonged infections, prolonged mpox was more commonly reported among people who were Black/African-American (23.9% vs 11.2%, p< 0.05, Table 1), those who were hospitalized (11.9% vs 4.3%), and PWH (59.7% vs 41.5%) (both p< 0.05, Table 2). No prolonged infections occurred in people who received two JYNNEOS doses. Among PWH, prolonged infections were more commonly reported among people with lower (< 200) CD4 counts (52.6% vs 6.0%) and higher (200+) HIV viral loads (62.5% vs 27.5%) (both p< 0.05, Table 3).

**Table 3.**
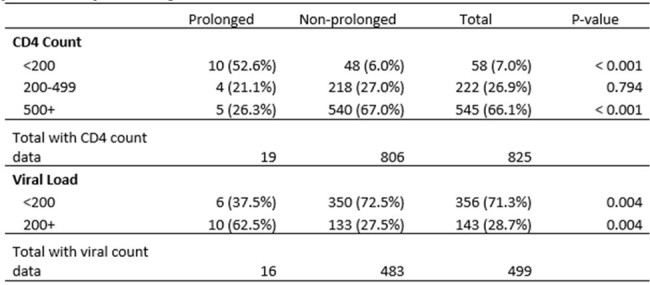
Mpox infection in California among people with HIV (N=2209) by CD4 count and viral load among PWH with laboratory data (CD4, n = 825; viral load, n = 499) within 1 year of mpox diagnosis

* Pair-wise comparisons were conducted comparing prolonged to non-prolonged for each clinical factor using Fisher’s Exact Test.

**Conclusion:**

Prolonged mpox infections were rare, but more commonly observed among people who identified as black or had HIV, especially if HIV was advanced. These groups, who may have disparities in health care access, should be prioritized for mpox vaccine education and outreach. Importantly, the detection of prolonged infections among these groups may have been facilitated through unmeasured confounders, such as access to care or other social determinants of health. While PWH were disproportionately represented among prolonged infections, over a third of prolonged cases occurred among people who were immunocompetent. Clinicians should be aware that mpox infection may occasionally persist for > 28 days and should consider retesting for mpox in people with prolonged symptoms, where initiation or change in antiviral therapy may be warranted.

**Disclosures:**

All Authors: No reported disclosures

